# Survival Prediction Using Transformer-Based Categorical Feature Representation in the Treatment of Diffuse Large B-Cell Lymphoma

**DOI:** 10.3390/healthcare11081171

**Published:** 2023-04-19

**Authors:** Sudarshan Pant, Sae-Ryung Kang, Minhee Lee, Pham-Sy Phuc, Hyung-Jeong Yang, Deok-Hwan Yang

**Affiliations:** 1 Department of Artificial Intelligence Convergence, Chonnam National University, Buk-gu, Gwangju 61186, Republic of Korea; 2 Department of Nuclear Medicine, Chonnam National University Medical School and Hwasun Hospital, Hwasun 58128, Republic of Korea; 3 Department of Hematology–Oncology, Chonnam National University Medical School and Hwasun Hospital, Hwasun 58128, Republic of Korea

**Keywords:** diffuse large B-cell lymphoma, prognosis, survival time prediction, deep learning, transformers

## Abstract

Diffuse large B-cell lymphoma (DLBCL) is a common and aggressive subtype of lymphoma, and accurate survival prediction is crucial for treatment decisions. This study aims to develop a robust survival prediction strategy to integrate various risk factors effectively, including clinical risk factors and Deauville scores in positron-emission tomography/computed tomography at different treatment stages using a deep-learning-based approach. We conduct a multi-institutional study on 604 DLBCL patients’ clinical data and validate the model on 220 patients from an independent institution. We propose a survival prediction model using transformer architecture and a categorical-feature-embedding technique that can handle high-dimensional and categorical data. Comparison with deep-learning survival models such as DeepSurv, CoxTime, and CoxCC based on the concordance index (C-index) and the mean absolute error (MAE) demonstrates that the categorical features obtained using transformers improved the MAE and the C-index. The proposed model outperforms the best-performing existing method by approximately 185 days in terms of the MAE for survival time estimation on the testing set. Using the Deauville score obtained during treatment resulted in a 0.02 improvement in the C-index and a 53.71-day improvement in the MAE, highlighting its prognostic importance. Our deep-learning model could improve survival prediction accuracy and treatment personalization for DLBCL patients.

## 1. Introduction

Diffuse large B-cell lymphoma (DLBCL) is the most common subtype of lymphoma, where tumors develop from lymphocytes, and comprises approximately one-third of non-Hodgkin’s lymphomas, which account for 90% of all lymphomas [1]. In addition, DLBCL is more likely to be diagnosed at an advanced stage and in older individuals compared to Hodgkin’s lymphomas [2]. Accurate prognosis prediction remains challenging regardless of the advances in treatment, with patients exhibiting diverse outcomes even within the same risk group. Despite standard therapy, 30–40% of DLBCL patients eventually relapse or are refractory to the initial immunochemotherapy [3]. Recently, novel therapeutic agents such as chimeric antigen receptor T cells and bispecific antibodies have been actively investigated for more effective and safer treatments in non-Hodgkin’s lymphoma patients [4].

Accurate prediction of prognosis and treatment outcomes can guide treatment decisions and enhance clinical trial designs [5]. Traditional survival analysis aims to identify the key covariates contributing to event occurrences such as death or relapse. The international prognostic index (IPI) is a well-established prognostic tool developed in 1993 [6] using pretreatment clinical risk factors including age, stage, lactate dehydrogenase (LDH), performance status, and extranodal involvement. However, individual patient’s treatment outcomes and prognoses have been revealed to be heterogeneous even in the same IPI risk group [7].

The Deauville score (DS) is a strong prognostic factor that is used to interpret F-18 fluorodeoxyglucose (FDG) positron-emission tomography (PET)/computed tomography (CT) imaging. Staging FDG PET can play a critical role in staging and risk stratification as it can identify the disease’s extent and location, including the involvement of extranodal sites and bone marrow. In addition, FDG PET/CT has played a crucial role in the prognostication of DLBCL patients. Interim FDG PET scans performed during treatment are used to assess chemosensitivity and predict prognoses [8], and the DS has been revealed to be predictive of patient outcomes [7,8,9,10].

Artificial intelligence has recently emerged as a promising tool to improve prognostic accuracy by leveraging large-scale clinical data and incorporating complex interactions among clinical, molecular, and imaging features. In medicine, the Cox proportional hazard (CPH) model [11], which is a semiparametric approach for calculating the hazard risk of the occurrence of an event, is the traditional standard method for survival analysis [11,12,13]. The CPH model assumes linearity among covariates, and several learning-based methods have been proposed to find non-linear relationships between various features. Machine learning methods such as random survival forests (RSF) [14], oblique random survival forests (ORSF) [15], and hazard boosting [16] have been successfully implemented in survival analysis. Researchers have employed Bayesian networks with the CPH model to improve its prediction performance and interpretability [17,18]. Faraggi et al. [19] extended the CPH model to include non-linearity using a multi-layer perceptron (MLP); however, although a non-linear approach was employed, this model failed to outperform the CPH model [20,21]. Artificial intelligence has allowed deep neural networks to efficiently learn key features from clinical data for survival prediction. Recent deep-learning approaches such as DeepSurv [22], CoxTime [23], and CoxCC [23] could replace the linear predictor with deep feed-forward neural networks to enable rich feature representation. DeepSurv demonstrated better performance than CPH on the concordance index (C-index) to model the interactions among covariates for treatment recommendation. The CoxTime model lifted the proportionality constraint by allowing time-dependent effects. Similarly, CoxCC is a proportional version of the CoxTime model. Introducing non-linearity enables the handling of more complex relationships between the clinical covariates and the survival times.

This study aims to develop a robust survival prediction strategy to integrate various risk factors effectively, including clinical risk factors and the DS, at different treatment stages. We conducted the experiments separately for the covariates obtained before and during treatment. Clinical information such as age can be treated as a continuous value, whereas variables such as stage and the DS are categorical. Despite being categorical, these classes cannot be considered purely independent because their order indicates the disease severity. For example, cancer stages (I, II, III, and IV) are assigned based on severity. Similarly, a DS of 1, 2, or 3 is less severe than a score of 4 or 5. As these values are neither continuous nor purely categorical, we consider them categorical variables and use transformer-based neural networks to capture the inter-class relationship within the categories.

Herein, motivated by the success of transformers in capturing critical features in various areas including natural language processing [24] and vision-related tasks [25], we design a transformer-based time-dependent survival model (TTSurv) for predicting the overall survival time. We follow the time-dependent approach of CoxTime and enhance the survival prediction using robust features learned through transformers. We compare the effectiveness of various clinical features obtained at two stages in the treatment process using various survival analysis methods. Based on the features’ time of availability, we divide them into before- and during-treatment groups. We then conduct experiments with the features in each group and evaluate the survival models using the C-index and the mean absolute error (MAE); the key contributions of this work can be summarized as follows:

- We design a systematic analysis of the clinical covariates based on their occurrence during various stages of the treatment.
- We propose a deep-learning-based method for predicting survival time in patients with lymphoma that leverages categorical embedding to represent the disease severity information in categorical data.

This paper is organized as follows. Section 2 describes the proposed method for survival prediction and Section 3 details the dataset, experiment design, and evaluation criteria used in this study. Section 4 provides the survival analysis results and a comparison with existing survival prediction methods. Section 5 presents the observations and discusses the further directions and challenges in survival analysis research. Finally, Section 6 concludes this paper.

## 2. Proposed Method

**Figure 1:**
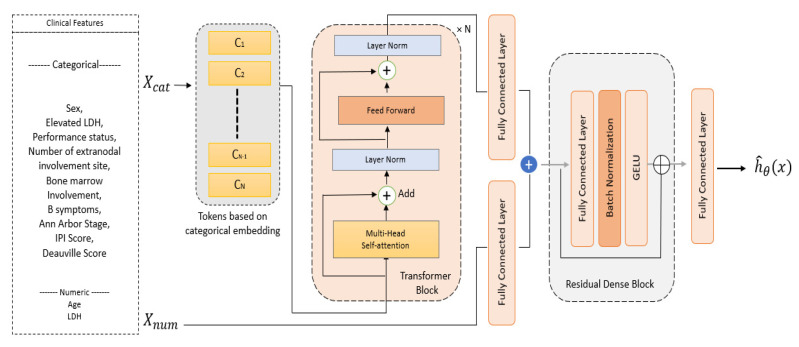
Architecture of the proposed model.

The MLP-based survival models typically use a shallow architecture with a few layers, which limits their ability to learn contextual information. Clinical features, $X=\{X_{cat},\;X_{cont}\},$ comprise both continuous and categorical features. Categorical features in medical data usually contain information related to severity; thus, we aim to learn the relationship between various classes in the categorical data by using transformers. To learn the categorical context, we designed TTSurv—a transformer-based time-dependent model with robust categorical representation (as illustrated in Figure 1). The dual-input model consists of two input branches: one for continuous features and one for categorical features. The proposed model’s main components are discussed below.

### 2.1. Categorical Embedding

Tabular data do not have a sequential context; hence, we replace positional encoding by following the column embedding method in Huang et al. [26] where each categorical feature $x_{n}$ in $X_{cat}=\left\{x_{1},x_{2},\ldots .x_{N}\right\}$ for $n\;\in \left\{1,\;2,\;\ldots .N\right\}$ is embedded into a learned embedding $E_{\emptyset }\left(x_{n}\right)$ with dimension d. The embedding for $x_{n}$ with c number of classes is generated by adding t special tokens such that the number of embeddings is n×c+t, which allows the model to distinguish between classes among various categorical features. The embeddings are passed through a series of transformer layers to extract important contextual features from the categorical data.

### 2.2. Transformer Encoders

Transformers [24] consist of multi-head self-attention layers, a multi-layer perceptron layer (MLP), layer normalization, and residual connections. A self-attention layer consists of the query (*Q*), key (*K*), and value (*V*) matrices defined as $Q\;\in {\mathbb{R}}^{m\times k}$, $K\;\in {\mathbb{R}}^{m\times k}$, and $V\;\in {\mathbb{R}}^{m\times v}$, respectively, where *m* is the number of embeddings passed to the transformer and *K* and *V* are the dimensions of the key and value vectors, respectively. The attention head is computed by $A\left(Q,K,V\right)=softmax\left(\left(QK^{T}\right)/\surd K\right).V$. The multi-head self-attention operation is followed by layer normalization and the MLP layer, and the multi-head self-attention helps learn context-aware features in a transformer.

### 2.3. Survival Prediction

The categorical features obtained from the transformer layers are concatenated with the numerical features and the combined feature, $concat\left(X_{embed}^{\prime }+C_{cont}\right)$, which is an input residual dense block for the survival prediction task. We follow the time-dependent approach in [23], where the time-dependent relative risk function is given as follows:

(1) $$h\left(t|x\right)=h_{0}\left(t\right).exp\left[g\left(t,x\right)\right].$$

We use the time-dependent version of the Cox partial likelihood function to optimize the model given by

(2) $$L_{NLL}=-\frac{1}{n}\sum_{i=1}^{n}\left(d_{i}*\log\left[f\left(t_{i}|x_{i}\right)\right]+\left(1-d_{i}\right)*\log\left[S\left(t_{i}|x_{i}\right)\right]\right),$$

where $x_{i},\;d_{i},$ and $t_{i}$ are the input feature, event indicator, and observed time, respectively, for patient i; and $f\left(t_{i}|x_{i}\right)$ and $S\left(t_{i}|x_{i}\right)$ are the density and survival functions at time $t_{i}$ for input $x_{i}$. We can find the cumulative hazard function from the predicted risk (1) using the Breslow estimator [27] to estimate the required survival function.

## 3. Experiments

### 3.1. Datasets

**Figure 2:**
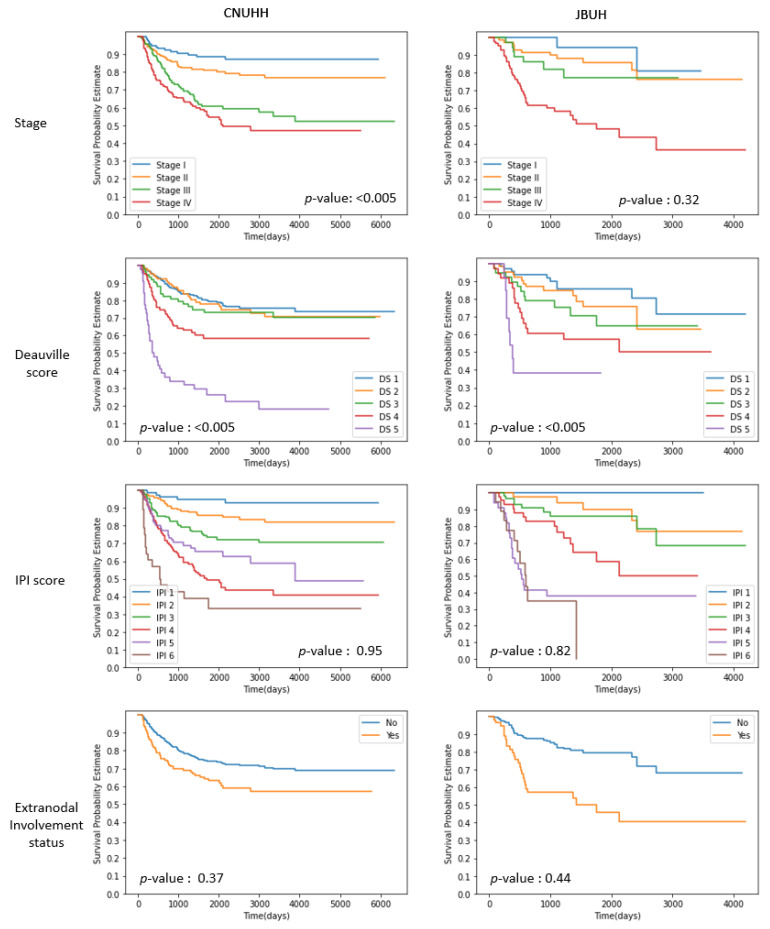
Kaplan–Meier plots of overall survival according to the stage, the Deauville score, the international prognostic index (IPI) score, and extranodal involvement status in the CNUHH and JBUH datasets.

We conducted experiments on two clinical datasets collected at Chonnam National University and Hwasun Hospital (CNUHH, *n* = 604) and Jeonbuk National University Hospital (JBUH, *n* = 220) in 2011–2018. This study was approved by the Institutional Review Boards of CNUHH (CNUHH-2022-095) and JBUH (CUH 2022-11-013). The log-rank test of the patient covariates in the datasets was statistically significant (*p* < 0.005), suggesting the importance of the individual features. Figure 2 depicts the Kaplan–Meier plots of patient properties.

The CNUHH and JBUH datasets have similar percentages of censored cases (70.86% and 73.18%, respectively). The clinical data consist of clinical information including patient age, sex, performance score, lactose dehydrogenase (LDH) level, stage, number of extranodal sites, presence of B-symptoms, and the IPI score. Additionally, for on-treatment evaluation, we included the DS calculated by experienced nuclear medicine physicians at CNUHH and JBUH through observations of interim PET scans.

The IPI prognostic tool was developed in 1993 using five significant risk factors (age, stage, LDH, performance status, and extranodal involvement). Table 1 shows the dataset’s characteristics before and during treatment. Only age and LDH (IU/L) in the dataset were used as continuous variables; all other covariates were considered categorical. The age distribution of the patients included in this study exhibits notable variance between the CNUHH and JBUH datasets. The former is composed of individuals aged 36–81 years, while the latter encompasses a wider age range of 15–87 years. This discrepancy can be attributed to the distinct patient populations treated at each institution during the period under investigation. Table 1 presents the age, sex, LDH, performance score, number of extranodal sites involved, bone marrow involvement, B symptom, Ann Arbor stage, the IPI score, and the DS before and during treatment.

### 3.2. Experiment Design

We conducted survival analysis experiments separately for pretreatment and on-treatment analysis based on the clinical information available before and during treatment. Only the DS was included as an additional feature for on-treatment analysis, which allowed us to compare the impact of the DS on survival prediction. We conducted experiments using the proposed method and compared it with existing state-of-the-art deep-learning survival models: DeepSurv, CoxCC, and CoxTime.

We evaluated the proposed model with five-fold cross-validation. To evaluate using cross-institutional data, we used clinical data from CNUHH and JBUH. First, the CNUHH dataset was split into training and validation sets using stratified k-fold sampling. The same set of training and validation data was used for different models to ensure a fair comparison. After the models were trained on the CNUHH dataset, we evaluated the trained models on the JBUH dataset, which was kept separate from the training process. The best weights saved at each fold were used for inference with the testing set and the average of each fold was reported. We implemented the proposed model in Python using PyTorch, and we used the implementation from the pycox library available on GitHub as the baseline model. The models were trained using an Nvidia GeForce RTX 3080Ti GPU with 12 GB of memory.

### 3.3. Evaluation Metrics

We evaluated the survival models using two performance metrics, namely the C-index and the MAE.

#### 3.3.1. The C-Index

The C-index is the most common evaluation method for survival analysis and is a measure of ranking for the predicted time. It estimates the probability that the predicted times for individuals and their true survival times have the same order, and is calculated as follows:

(3) $$C-\mathrm{index}=\frac{\Sigma_{i,j}\;1_{T_{j}<T_{i}}\;.\;1_{\eta_{j}>\eta_{i}}.\delta_{j}}{\Sigma_{i,j}\;1_{T_{j}<T_{i}}\;.\delta_{j}},$$

where $\eta_{i}$ represents the risk score of a unit. In addition, $1_{T_{j}<T_{i}}$ is 1 when $T_{j}<T_{i}$ and 0 otherwise, and $1_{\eta_{j}>\eta_{i}}$ is 1 when $\eta_{j}>\eta_{i}$ and 0 otherwise. A C-index of 1.0 indicates perfect concordance and 0.5 represents poor prediction.

#### 3.3.2. The MAE

Although the C-index measures the accuracy of ranking the survival times, it may not provide a fair assessment of a model’s overall performance. For example, the C-index does not consider the magnitude of the difference between predicted and actual survival times. We address this limitation by using the MAE as an additional evaluation metric for survival time prediction models. The MAE involves converting predicted hazards into survival functions and calculating the average difference between the predicted residual life and the true survival time. However, it cannot be used for all samples due to the presence of censored data. We evaluated the MAE for patients with observed events based on each patient’s median life. The MAE, as a complementary metric, provides additional comparison criteria when true survival times are known, and is calculated as follows:

(4) $$\mathrm{MAE}=\frac{1}{N_{E=1}}\sum_{i=0}^{n}N_{E=1}\times \left|y_{i}-{\hat{y}}_{i}\right|,$$

where $N_{E=1}$ represents the number of samples that observed the event and $y_{i}$ and ${\hat{y}}_{i}$ represent the true and predicted survival times, respectively, for the ith sample.

## 4. Results

We compared the performance of the proposed model with DeepSurv, CoxTime, and CoxCC; similar performance was observed for all models. Table 2 and Table 3 present the experimental results on the CNUHH and JBUH datasets. The CNUHH column lists the average values of five-fold cross-validation. The model saved at each fold was used to evaluate the unseen data from the JBUH dataset. The proposed model outperformed the existing best-performing model on the CNUHH dataset while achieving a comparable C-index with existing survival analysis methods on the JBUH dataset. We evaluated the models using the C-index and the MAE.

**Figure 3:**
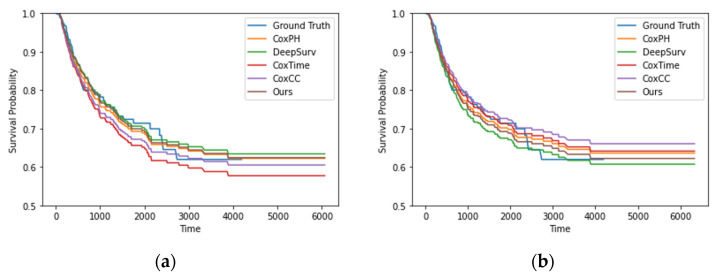
Survival curves for each model in the test set: (**a**) before treatment; (**b**) after treatment.

The proposed model was found to outperform existing models in terms of the MAE. Moreover, it performed better on unseen data, which suggests that the transformer architecture used in this model was effective at extracting robust features and enabling generalizability. Additionally, we observed that the models exhibited even higher performance when an additional feature obtained during treatment was included. This finding highlights the high prognostic importance of the DS in patients with DLBCL. Figure 3 presents the survival curves of the test set as obtained using various survival models. The estimated survival function was similar for various methods.

**Figure 4:**
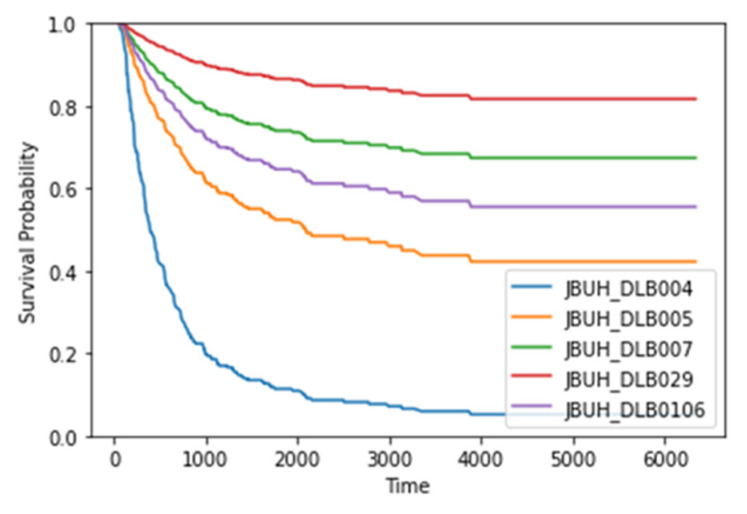
Representative estimated individual survival curves for five patients with ground truth survival times in the range of 527–2421 days.

Figure 4 provides the estimated survival plots for five patients with survival times in the range of 321–2127 days; Table 4 lists corresponding survival time predictions. The results revealed that the model could estimate the survival times with little error in terms of days. However, Patient JBUH_DLB106 had a low predicted survival time despite their actual survival time being similar to JBUH_DLB029. This outcome could be a special case where the patient lived longer despite having severe symptoms. Patient JBUH_DLB106 was at stage III with a low LDH of 756 and a Deauville score of 3.

**Figure 5:**
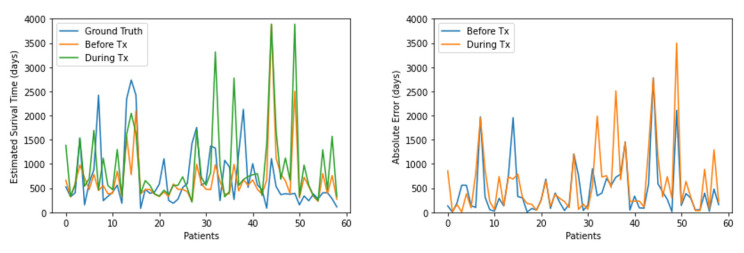
Estimated survival times and absolute error comparison between before and during treatment.

As indicated in Figure 5, we compared features used both before and during treatment analysis for all uncensored patients in the test set. The estimated survival times were generally close to the ground-truth values. However, the survival times were poorly estimated for some cases, as mentioned in Table 4. In summary, we demonstrated that TTSurv could estimate the survival times with a relatively small MAE of approximately 559 days.

## 5. Discussion

The proposed model, TTSurv, outperformed the existing state-of-the-art survival prediction model for patients with DLBCL using transformer-based deep-learning models regarding the C-index and the MAE in the dataset. Therefore, we have demonstrated the potential of deep-learning models to reliably predict survival times based on clinical features and the DS. In addition, we have illustrated the importance of the DS obtained during treatment, which significantly improved the model performance, indicating this feature’s high prognostic value. We conducted survival analyses based on two stages: before and during treatment. Although most prognostic clinical features were available at the beginning of treatment, the DS was only available after the interim-PET scan. Our results add to the growing body of evidence supporting the high prognostic value of the DS [7,8,9,10].

Deep-learning models have demonstrated usefulness in interpreting clinical data and providing better prognosis predictions with less manual feature engineering. Manual feature selection is a traditional method of analyzing clinical data that may not capture all pertinent information. The feature selection process requires expert knowledge and may result in a limited view of the data, resulting in incomplete or inaccurate conclusions. In addition, clinical data often contain categorical features that are not purely independent classes; these features may have complex interrelationships with other variables and their treatment as independent classes may result in the loss of valuable prognostic information. Therefore, a more sophisticated approach is required to ensure that all relevant information is considered and that categorical features are appropriately processed to provide more accurate predictions. We used transformer-based categorical data encoding on clinical datasets to address this problem and developed a deep-learning network for survival prediction.

Moreover, clinical data features are typically grouped into numeric and categorical data. However, the categories featured in clinical data do not have purely independent classes. For instance, patients with cancer are usually categorized into four stages upon diagnosis: I, II, III, and IV. As such, data are not continuous and they are often treated as categorical even though they carry information related to the disease severity on an ordinal scale, and this may result in a loss of valuable information. As transformers have been widely accepted in various domains, including natural language processing and vision-related tasks, we adopted transformers to encode categorical features using transformers. TabTransformer has demonstrated high performance in handling tabular data. Therefore, we adopted transformer-based categorical data encoding in clinical datasets and developed a deep learning network for survival analysis.

Survival analysis is considered more challenging than standard regression tasks due to the presence of data censoring. Censoring occurs when the event of interest is not observed during the study period for various reasons, such as subjects leaving before this study is complete or this study finishing before the event of interest occurs. For example, in survival analysis where the event of interest is death, some patients who survive until the end of the study duration may opt out or move to a different hospital during this study. Survival models manage information censoring by including an event indicator, which is a binary variable that indicates whether the event occurred during the study period. However, data censoring has the potential to reduce model accuracy. In future research, we plan to use larger clinical datasets that include numerous non-censored data to increase our model’s performance.

While the proposed transformer-based survival prediction model has shown promising results in predicting patient outcomes, it is important to acknowledge its limitations regarding its practicability in clinical contexts. One major limitation is that the model relies solely on clinical information and the DS calculated by the experts, which may not capture all relevant clinical information such as imaging information in PET/CT. In our future studies, we plan to overcome this limitation by incorporating radiological images to improve the model accuracy. Additionally, we aim to validate the model on a larger and more diverse patient population to ensure its generalizability. Despite these limitations, our transformer-based model represents a significant step forward in the field of predictive analytics in healthcare and holds great potential for improving patient outcomes.

## 6. Conclusions

This study reveals the potential of using a transformer-based deep-learning model for survival prediction in patients with DLBCL. We demonstrated the importance of incorporating the DS obtained during treatment and the effectiveness of using categorical embedding in handling high-dimensional and categorical clinical data. While the model outperformed existing state-of-the-art survival models, we acknowledge the need for larger clinical datasets and the inclusion of more prognostic modalities to increase the model’s performance. This study suggests that deep-learning models may improve personalized treatment and survival prediction accuracy for patients with DLBCL.

## Figures and Tables

**Table 1:** Patient characteristics at different treatment stages.

Time of Evaluation	Characteristics		CNUHH (*n* = 604)	JBUH (*n* = 220)
Pretreatment evaluation	Age (years)		36–81	15–87
	Sex	Female	250	88
		Male	354	132
	LDH (IU/L)		144–8402	244–3797
	LDH (normal vs. elevated)	Elevated	314	132
		Normal	290	88
	ECOG performance status	1	323	190
		2	201	16
		3	66	11
		4	14	3
	Number of extranodal involvement sites	0	166	73
		1	291	93
		2	120	30
		3	20	11
		4	3	7
		5	3	4
		6	1	2
	Bone marrow involvement	Yes	48	46
		No	556	174
	B symptoms	Yes	99	58
		No	505	162
	Ann Arbor stage	I	118	23
		II	196	73
		III	137	37
		IV	153	87
	IPI score	0	81	29
		1	152	39
		2	135	58
		3	131	43
		4	77	33
		5	28	18
On-treatment evaluation	Deauville score	1	290	67
		2	108	65
		3	87	39
		4	75	36
		5	44	13

ECOG: Eastern Cooperative Oncology Group, IPI: international prognostic index, and LDH: lactate dehydrogenase.

**Table 2:** Concordance index for overall survival prediction.

	CNUHH		JBUH	
	Before	During	Before	During
CoxPH [11]	0.7134	0.7440	**0.7858**	**0.7990**
DeepSurv [22]	0.7213	0.7428	0.7403	0.7546
CoxCC [23]	0.6925	0.7055	0.7407	0.7501
CoxTime [23]	0.6929	0.7358	0.7384	0.7782
TTSurv	**0.7245**	**0.7457**	0.7756	0.7950

The results for the best performing models are denoted in bold font.

**Table 3:** Mean absolute error for overall survival prediction.

	CNUHH		JBUH	
	Before	During	Before	During
CoxPH [11]	1092.8914	969.0686	822.1458	662.0441
DeepSurv [22]	1117.5200	1006.5200	911.6237	873.2576
CoxCC [23]	1047.7943	1023.1200	798.5797	783.5695
CoxTime [23]	1195.6286	1011.7200	915.0136	774.6542
TTSurv	**995.3200**	**958.0857**	**613.5119**	**559.8000**

The results for the best performing models are denoted in bold font.

**Table 4:** Example predictions for patients with survival times in the range of 527–2421 days.

Patient ID	Ground Truth (Days)	Median Life (Days)	
		Before Tx	During Tx
JBUH_DLB004	321	324	332
JBUH_DLB005	403	600	564
JBUH_DLB007	1537	977	1531
JBUH_DLB029	2340	1571	1611
JBUH_DLB106	2127	673	677

## Data Availability

The data used in this research are not publicly available.
